# Evaluation of biolistic gene transfer methods in vivo using non-invasive bioluminescent imaging techniques

**DOI:** 10.1186/1472-6750-11-62

**Published:** 2011-06-02

**Authors:** Jixiang Xia, Angela Martinez, Henry Daniell, Steven N Ebert

**Affiliations:** 1 Burnett School of Biomedical Sciences, University of Central Florida College of Medicine, Orlando, FL 32827 USA

**Keywords:** Bioluminescence, Gene Therapy, Biolistic, Mice, Imaging, Non-invasive

## Abstract

**Background:**

Gene therapy continues to hold great potential for treating many different types of disease and dysfunction. Safe and efficient techniques for gene transfer and expression in vivo are needed to enable gene therapeutic strategies to be effective in patients. Currently, the most commonly used methods employ replication-defective viral vectors for gene transfer, while physical gene transfer methods such as biolistic-mediated ("gene-gun") delivery to target tissues have not been as extensively explored. In the present study, we evaluated the efficacy of biolistic gene transfer techniques in vivo using non-invasive bioluminescent imaging (BLI) methods.

**Results:**

Plasmid DNA carrying the firefly luciferase (LUC) reporter gene under the control of the human Cytomegalovirus (CMV) promoter/enhancer was transfected into mouse skin and liver using biolistic methods. The plasmids were coupled to gold microspheres (1 μm diameter) using different DNA Loading Ratios (DLRs), and "shot" into target tissues using a helium-driven gene gun. The optimal DLR was found to be in the range of 4-10. Bioluminescence was measured using an In Vivo Imaging System (IVIS-50) at various time-points following transfer. Biolistic gene transfer to mouse skin produced peak reporter gene expression one day after transfer. Expression remained detectable through four days, but declined to undetectable levels by six days following gene transfer. Maximum depth of tissue penetration following biolistic transfer to abdominal skin was 200-300 μm. Similarly, biolistic gene transfer to mouse liver in vivo also produced peak early expression followed by a decline over time. In contrast to skin, however, liver expression of the reporter gene was relatively stable 4-8 days post-biolistic gene transfer, and remained detectable for nearly two weeks.

**Conclusions:**

The use of bioluminescence imaging techniques enabled efficient evaluation of reporter gene expression in vivo. Our results demonstrate that different tissues show different expression kinetics following gene transfer of the same reporter plasmid to different mouse tissues in vivo. We evaluated superficial (skin) and abdominal organ (liver) targets, and found that reporter gene expression peaked within the first two days post-transfer in each case, but declined most rapidly in the skin (3-4 days) compared to liver (10-14 days). This information is essential for designing effective gene therapy strategies in different target tissues.

## Background

Gene therapy is a promising strategy for correcting both genetic and acquired diseases [1,2]. There are a variety of gene delivery methods currently available, with the main purpose of any given strategy being to efficiently transfer and express the target gene(s) of interest without adverse side-effects. Development of improved gene delivery strategies is critical for application of effective gene therapies, yet evaluation of the effectiveness of these strategies is often hampered by difficulties in detecting transgene expression in real-time in vivo. Non-invasive bioluminescent imaging (BLI) has been successfully used for evaluation of cell and gene therapies in small animal models [3-6], though it has not yet been systematically applied to analysis of physical gene transfer methods. The purpose of the present study is to evaluate the effectiveness of biolistic gene transfer in different types of tissue in mice using non-invasive in vivo bioluminescence imaging (BLI).

Vectors for gene delivery can be divided into two general categories: viral and non-viral. Viral vectors are widely employed for gene therapy as they are highly efficient, and the effects can be sustained over periods of weeks, months, and sometimes years [7,8]. A number of different viral vectors have been utilized for gene therapy approaches based on cell/tissue-type preferences. Some of these include adeno- and adeno-associated virus, herpes virus, retrovirus, and lentivirus, among others [7,8]. Each has its own advantages and disadvantages depending on application and tissue type. In general, recombinant replication-defective (ie, crippled) viral vectors are used for this purpose to minimize threats of infection, immune responses, and other potentially adverse conditions. In the past, serious consequences have resulted from the use of viral vectors for gene therapy in humans [9,10]. Most notably, the death of 18-year old Jesse Gelsinger due to immune complications arising in response to adenovirus vectors essentially halted clinical trials using viral vectors for gene therapy for many years [11-13]. More recently, other viral vector-based gene therapy approaches have come into question because of associated genomic instability and activation of proto-oncogenes [14,15]. Thus, it is becoming increasingly clear that there is an inherent risk of serious adverse side effects with some viral-based gene therapy strategies.

Alternatively, non-viral gene therapy strategies pose less risk of infection and/or adverse immune responses. Non-viral gene transfer techniques typically involve either chemical or physical methods. Chemical methods such as calcium-phosphate or liposome-based approaches have achieved much success for in vitro applications in cell cultures, but have been of more limited utility in vivo due to low transfection efficiencies and toxicity issues [16-18]. Physical methods are diverse and include direct injection of DNA, electroporation, ultrasonic-based, and biolistic approaches in addition to a variety of other techniques [16-18]. In most cases where chemical or physical methods are employed, purified plasmid DNA is used for delivery. When effective, this typically results in transient expression in target tissues since the "naked" DNA is eventually degraded by host nucleases [19]. Thus, these methods are currently limited to specific applications where transient expression of a transgene is warranted (*e.g*., induction of angiogenesis) [20-23].

In the present study, we chose to use physical methods of gene transfer to avoid the complications associated with viral and chemical strategies. In particular, we explore the use of biolistic methods of gene transfer due to its widespread applicability and low toxicity. Biolistic gene transfer has been used for many years primarily for the study and production of transgenic plants [24-27]. It is, in fact, the preferred and most commonly used method for gene transfer in plants due to its versatility and effectiveness. Due to their tough outer cell walls, plants typically require helium pressures well in excess of 1000 pounds/square inch (psi), and the procedure is usually performed under vacuum using a stationary biolistic gene delivery chamber. Animal cells, in contrast, cannot tolerate bombardment with such high pressure, nor are they amenable to vacuum conditions for the transfer process. In recent years, however, a hand-held device known as the Helios™ gene gun (Bio-Rad Labs, Hercules, CA) has been developed for biolistic gene transfer experiments in animals using lower helium pressures (≤ 600 psi) [28]. No vacuum is required, and the DNA-gold particles can be delivered from a hand-held gun that can be used to target virtually any tissue or organ for direct biolistic gene transfer. This device has been used to successfully perform biolistic gene transfer in a number of mammalian cell-culture and live-animal models [29-33]. In the current report, we use BLI to evaluate the effectiveness of gene transfer via biolistic techniques in vitro and in vivo.

## Methods

### Gene Gun Materials

The hand-held Helios Gene Gun, Tubing Prep Station, Optimization Kit (including gold microcarriers, polyvinylpyrrolidone or PVP, and tubing), and related supplies were obtained from Bio-Rad, Inc. (Hercules, CA).

### Reagents

Human embryonic kidney (HEK) 293 cells and mouse embryonic stem cells were maintained as previously described [6,34]. Cell culture reagents including Dulbecco's Modified Eagle Medium (DMEM) and supplements were obtained from Invitrogen, Inc. (Carlsbad, CA). Fetal bovine serum was purchased from Hyclone Labs (Logan, UT). Lipofectamine 2000 and XGAL were obtained from Invitrogen, Inc (Carlsbad, CA). Luciferin for in vivo use was obtained from Caliper Labs (Hopkinton, MA). Bright-Glo™ Luciferase Assay Kit for in vitro assays was supplied by Promega (Madison, WI). All other chemicals and other reagents used in this study were obtained from Sigma-Aldrich (St. Louis, MO).

### Plasmids

Plasmids used in this study included pCMV-LUC (Clontech, Menlo Park, CA), pNCX1-LUC [35], and pCMV-beta-galactosidase (pCMV-βGal) [36] have been described previously as indicated. All plasmids were purified using Qiagen Maxi-Prep DNA purification kits (Valencia, CA) followed by phenol:chloroform:isoamyl alcholol (25:24:1) extraction, ethanol precipitation, 70% ethanol wash and air-drying. The dried pDNAs were then resuspended in Tris-EDTA (TE, pH 8.0) buffer at a concentration of 1 mg/ml. We found that the additional organic extraction and ethanol precipitation/washing steps were critical for achieving efficient coupling of pDNAs to gold microcarriers.

### Animals

Adult white FVB mice (18-25 g each) were used for this study. The mice were housed in the Transgenic Animal Facility at the University of Central Florida (UCF) on a 12:12 hr light:dark cycle, and provided food and water ad libitum. All procedures utilizing mice in this study were performed in accordance with approved UCF IACUC protocols consistent with NIH regulations governing vertebrate animal research.

### Preparation of gold microcarrier-coated cartridges

Preparation of gold microcarrier-coated cartridges was performed according to the manufacturer's instructions (Bio-Rad, Inc., Hercules, CA) as previously described [31,37]. Briefly, 25 mg of gold microcarriers (1 μm diameter average size) were suspended in absolute ethanol containing 0.05 M spermidine. An equal volume of pDNA was added to this mixture, vortexed, and sonicated. Various amounts of pDNA were used to evaluate different DNA-loading ratios (DLRs). By definition, DLR of 1 = 1 μg DNA per mg of gold microcarrier particles [37]. An equal volume of 1M CaCl_2 _was added to the mixture in dropwise fashion, and then precipitated at room temperature for 5 min (the volume of spermidine was always the same as those of plasmid and CaCl_2_). The solution was microcentrifuged (14,000 ×g) for 5s and the supernatant was removed. The resulting pellets were resuspended with 100% ethanol and washed three times with same for 15s each. Finally, the pellets were each resuspended in 2.5 ml absolute ethanol containing 0.05% polyvinylpyrrolidone (PVP), and sonicated to achieve uniform suspension of microcarrier particles prior to cartridge loading. Cartridge tubing was loaded into the Bio-Rad Tubing Prep Station, dried with nitrogen gas, and coated internally with the microcarrier suspension during continuous rotation of the tubing. After complete drying, the tubing was cut into 0.5 inch cartridge "bullets" using the supplied tubing cutter, and stored in the parafilm-sealed containers at 4°C until ready for use.

### Electrophoresis of microcarrier mixtures

Just before the last centrifugation step to concentrate the microcarriers in absolute ethanol (see preceding paragraph), a portion of the suspension was transferred to microcentrifuge tubes, and dried in a Speed-Vac centrifuge (Savant Instrument Inc, Farmingdale, NY). The pellet was resuspended in electrophoresis loading buffer and immediately subjected to electrophoresis in 0.8% agarose gels containing 0.2 μg/ml ethidium bromide. Each well was loaded with approximately equal amounts of microcarriers. The gels were imaged under ultraviolet light.

### In vitro gene delivery

For biolistic delivery of reporter genes, cells were trypsinized and transferred to six well plates and kept until 80% confluence prior to gene transfer. Immediately before transfection, the medium was gently removed and washed once with PBS, the barrel ring of hand held gene gun was centered at the well and the distance to the cells was about 2 cm. Upon pulling the trigger, the gold microcarriers were shot out of the cartridges by helium with pressures between 100-150 psi (1psi = 6.89 kPa). Fresh growth media was added to the dishes and the cells were recovered in the incubator for another 48 h before bioluminescence measurement. As a positive control, some wells were transfected in parallel using lipofectamine according to the manufacturer's instructions (Invitrogen, Inc.; Carlsbad, CA).

### In vivo gene delivery

Mouse skin and liver were chosen as the targets of bombardment for dynamic gene expression observation. Prior to the procedure, the mice were administered with 2% isoflurane to achieve a surgical plane, and maintained as such using a nose-cone for continuous isoflurane delivery (in oxygen, flow speed = 1L/min). The mice were placed in supine position on a surgical pad, and the abdominal hair was removed by Nair^® ^hair-removal lotion. For biolistic transfer to skin, the barrel ring of the Gene Gun was lightly touching the skin. For biolistic transfer to liver, an abdominal incision was made to expose the organ, and the barrel of the Gene Gun was positioned directly above the target. Following biolistic delivery, the incision was sutured, and the mice were administered buprenorphine (0.05 mg/kg) in the thigh muscle to help mediate pain or discomfort associated with the procedure. The mice were then removed from anesthesia, returned to their cages, and were ambulatory within a few minutes.

To determine if differences of DLR could affect expression of biolistically-transferred genes, the mice were divided into four groups with six mice per each group, and DLR was varied between 0, 4, 10 and 25. Helium pressure was set to 200 psi, and the microcarrier bombardment was targeted to abdominal skin. In a subsequent series of experiments, the helium pressure was adjusted to 300-400 psi for biolistic delivery to mouse skin since the lower pressure (200 psi) was well-tolerated in the initial group. For biolistic transfer to soft tissue (liver), helium pressure was held at a maximum of 200 psi to minimize tissue damage.

### Bioluminescence imaging (BLI)

For in vitro BLI, luciferase assays were performed as previously described [36] except that the results were quantified using an In Vivo Imaging System-50 (IVIS-50) from Caliper Labs (Hopkinton, MA). For in vivo BLI assessment, mice were injected i.p. with D-luciferin potassium salts (Caliper Life Sciences, Hopkinton, MA) at a dosage of 150 mg/kg, and maintained for 5 min before imaging. The mice were then anesthetized with 2% isoflurane and placed in the IVIS-50 chamber in supine position where they were maintained with isoflurane administered through nose-cone ports inside the chamber. The chamber temperature was kept constant at 37°C throughout the procedure. Light emission was collected for 5 min, and the intensity was represented as the number of photons per second/cm^2^/steradian for a designated "Region of Interest" or "ROI". A standard "ROI" template was used for each experiment so that direct comparison of different data sets could be readily managed. Images were processed using Living Image^® ^software (Caliper Life Sciences, Hopkinton, MA).

### XGAL histological staining

Following biolistic transfer of pCMV-βGAL to mouse skin as described above, mice were sacrificed three days later by decapitation while under full anesthesia (2% isoflurane). The abdominal skin surrounding the biolistic target area (22 mm diameter) was excised and fixed with 4% paraformaldehyde for 1-2 hrs on ice. The tissue was then transferred to a solution of 30% sucrose in PBS and kept at 4°C overnight. The skin was sectioned transversely (14 μm/section) using a cryostat instrument. The protocol for the XGAL staining with acidified eosin counterstaining was performed as described previously [38].

### Statistical analysis

Results are expressed mean values ± standard deviation. One-way analysis of variance was used to determine if statistically significant differences occurred, with p < 0.05 required to reject the null hypothesis.

## Results

**Figure 1:**
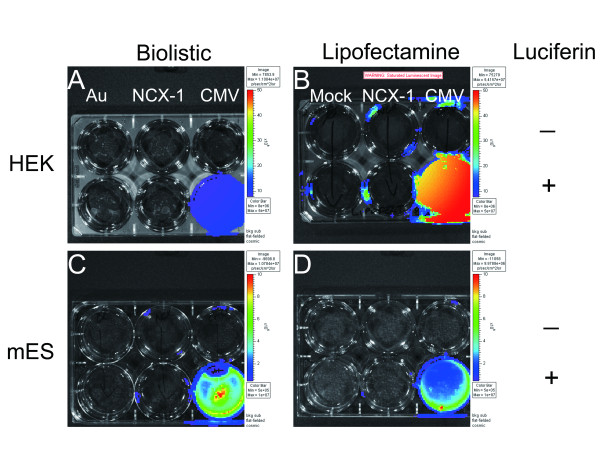
**Luciferase expression in transfected cells in culture**. (A&C) HEK 293 and (B&D) mouse ES cells transfected using either lipofectamine 2000 (A&B) or the Helios Gene Gun (C&D). The top row in all sets of plates shown did not receive the luciferin substrate whereas it was provided to all wells in the bottom row of each plate shown. The leftmost column of each plate contained mock-transfected cells. The middle column was transfected with pNcx1-LUC, and the rightmost column of each plate was transfected with pCMV-LUC.

Our initial experiments were designed to test the functionality of the biolistic gene transfer and BLI methods. To accomplish this, we bombarded two different cell types in culture with gold (Au) particles coated with or without LUC reporter plasmid DNAs driven by different promoters. As shown in Figure 1A, HEK cells blasted with gold particles coated with pCMV-LUC produced a strong bioluminescent signal in the presence of the luciferin substrate. In contrast, cells blasted with gold particles alone (Au) or coated with LUC reporter plasmids driven by the cellular promoter from the sodium-calcium exchanger 1 (NCX1) gene generated no measurable bioluminescent signal. In parallel, we performed an analogous transfection experiment using an established in vitro transfection method using lipofectamine [39]. The results were similar to those achieved with the biolistic method except that CMV-LUC activity was much more robust in the lipofectamine sample compared with the biolistic sample in HEK cells. In contrast, biolistic gene transfection produced brighter bioluminescence than lipofectamine for CMV-LUC in mES cells (Figure 1C and 1D). The pNCX1-LUC construct did not produce measurable bioluminescence when transfected by either method, and thus was not explored further in this study.

**Figure 2:**
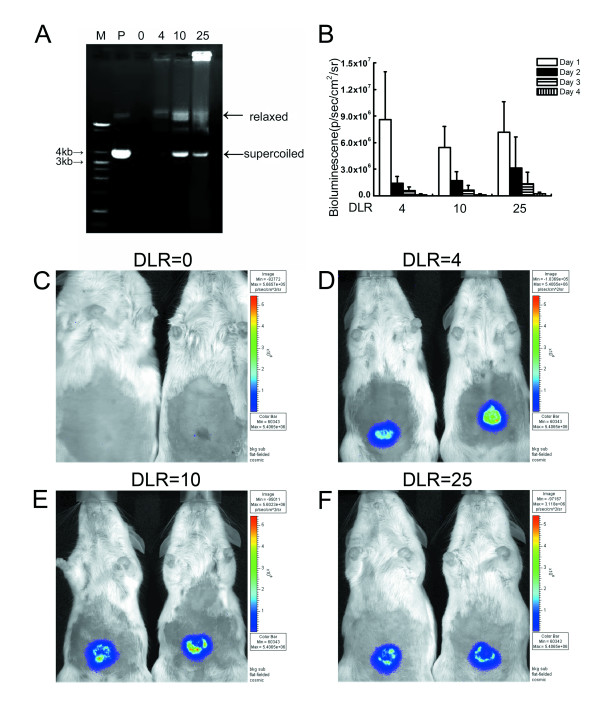
**Evaluation of DNA-Loading ratio (DLR)**. (A) Picture of ethidium bromide-stained agarose gel showing plasmid DNA-gold microcarrier coupling. The amount of microcarriers calculated to contain 100 ng of plasmid DNA was loaded per lane. Lanes: M, marker (1 Kb ladder; P, plasmid DNA (pCMV-LUC) alone; 0, 4, 10, 25 refer to DLRs for the respective lanes indicated. (B) Luciferase activity in mouse skin following biolistic transfer of pCMV-LUC at different DLRs. BLI was performed daily for four days after gene delivery (n = 6). (C-F) Representative pictures from different DLRs one day after biolistic gene transfer.

To perform biolistic transfer of LUC reporter plasmid DNA in vivo, we next evaluated the effectiveness of physical coupling of plasmid DNA to gold microcarrier particles. The gold microcarriers were 1 μm in diameter, on average, and increasing DNA loading ratios (DLRs) were evaluated by agarose gel electrophoresis. As shown in Figure 2A, increasing the amount of pCMV-LUC in the coupling reactions led to greater retardation of the plasmid through the gel, thereby indicating that more of the plasmid DNA was being coupled to the microcarriers. As the DLR increased from 4 to 10 or 25, however, increased amounts of uncoupled plasmid were observed ("supercoiled" and "relaxed" bands, arrows, Figure 2A), possibly indicating that some saturation of binding had occurred. When the DLR was increased from 10 to 25, most of the plasmid did not enter the gel presumably because higher order DNA-gold coupling had occurred such that the complexes were now too large to enter the gel. Less of the "free" supercoiled and relaxed plasmid DNA was present with a DLR of 25 compared to that observed with a DLR of 10. Macroscopic inspection revealed some "clumping" of gold particles at the highest DLR of 25, which is consistent with the idea that higher order coupling likely occurred in this group.

To evaluate the efficacy of these different DLRs for biolistic gene transfer, we employed the Helios gene gun to deliver the gold microcarriers into mouse skin in vivo. Reporter gene expression from the pCMV-LUC vector was then measured using BLI. Quantitative analyses of these results are shown in Figure 2B. Expression appeared highest 24 h after biolistic delivery, and then declined steadily over the next few days. Surprisingly little difference was observed with the different DLRs. Representative images of in vivo BLI for mice in these experiments are shown in Figure 2, panels C-F. In the absence of plasmid DNA, no BLI was apparent (DLR = 0, Figure 2C). In contrast, the BLI results for panels D-F (Figure 2) showed similar levels of bioluminescence activity under the conditions used for these experiments. No significant differences in bioluminescence were seen using microcarriers with DLRs = 4, 10, and 25, though there was a trend towards increased bioluminescence with increasing DLR after day one.

**Figure 3:**
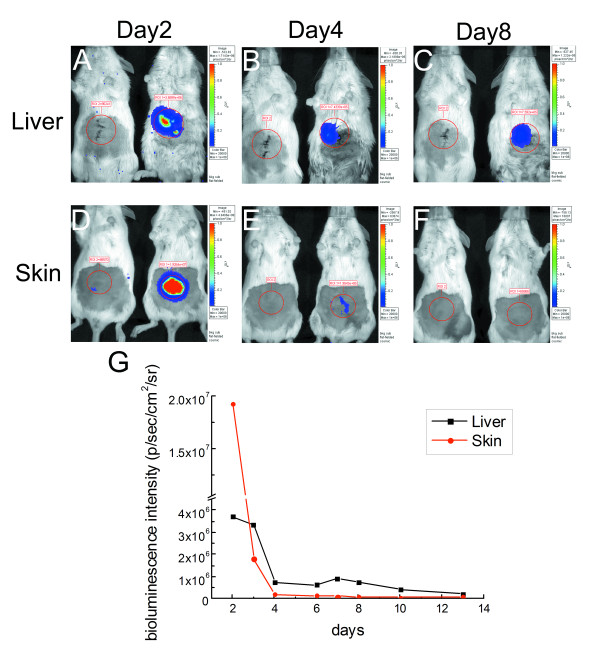
**Comparison of BLI following biolistic reporter gene transfer into mouse liver (A-C) or skin (D-F)**. Representative mice are shown at day 2 (A and D), 4 (B and E) and 8 for (C and F) following biolistic gene transfer. In each panel, the mouse on the left was transfected with gold microcarriers alone, and the mouse on the right was transfected with gold microcarriers conjugated with pCMV-LUC (DLR = 10). Quantitative assessment of these data is shown in panel G.

To maximize gene transfer effectiveness and consistency, we chose the middle DLR of 10 to compare the efficacy of biolistic gene transfer into different tissue types in vivo. In this series of experiments we used BLI to measure reporter gene activity following biolistic transfer into either superficial (skin) or internal (liver) tissue in vivo, as shown in Figure 3. Peak BLI activity was observed at the 2d time-point following biolistic gene delivery into both tissues, with skin showing much greater BLI activity than liver at this point. Over time, however, BLI activity in liver was sustained much longer than that seen in skin. For example, relatively strong BLI activity persisted through 8d after gene transfer into liver, whereas BLI activity in skin was nearly undetectable by the 8d time-point. BLI activity remained relatively stable in the liver between 4-8d following gene transfer, and then began to steadily decline over the next week (Figure 3G). The last time-point measured in these experiments was 13d post-delivery, and there was still a small but measurable amount of BLI activity present in the liver group. These results show that while biolistic gene transfer was effective for both liver and skin, the dynamic features of reporter gene expression in these two different tissues varied over time.

**Figure 4:**
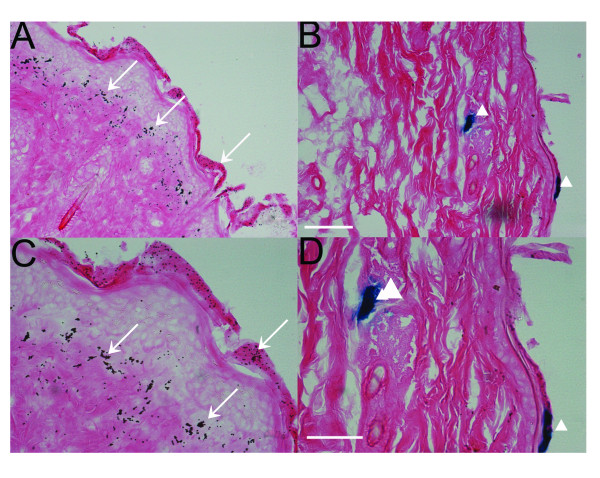
**Identification of transfected cells *in vivo *following biolistic delivery of gold-coupled pCMV-βGal into mouse skin**. (A-C) Low-magnification (20X objective; scale bar, 100 μm) and (D-F) Higher-magnification (40X objective; scale bar, 60 μm) views of transverse sections of mouse skin collected three days after biolistic gene transfer. The sections were stained with XGAL (blue) and eosin (pink). Examples of microcarriers are indicated by arrows, and transfected cells were identified by blue XGAL staining (arrowheads).

To determine the depth of gold microcarrier penetration following bombardment of mouse tissue, we performed histological assessments of abdominal skin 3d following biolistic gene transfer. In these experiments, we used pCMV-βGAL reporter plasmid (DLR = 10) to facilitate observation of reporter gene activity in histological sections. The gold microcarriers were readily observed in histological sections (Figure 4, arrows). Many of them were found in the outermost layer of skin (epidermis), but there were clearly clusters of these particles found in deeper layers. Most of these particles were found approximately 100 μm from the surface of the skin, though some were observed as deep as 200-300 μm from the surface. In contrast, reporter gene activity, as visualized by blue XGAL staining, was mainly found near the surface of skin, though patches of cells expressing βGAL were seen as deep as ~150 μm from the skin surface (Figure 4, arrowheads). These results indicate that biolistic gene transfer was effective at or near the tissue surface under the conditions employed in these experiments.

## Discussion

In the present study, we have shown that different types of tissues display differential kinetics of transgene expression following physical gene transfer in vivo. We evaluated biolistic gene transfer in external (skin) and internal (liver) tissue. In each case, we were able to effectively monitor and quantify reporter gene expression using BLI. The main advantage of this approach is that expression of the gene transferred could be evaluated repeatedly in the same animals over a period of several days using non-invasive imaging (BLI) methods. Thus, this strategy requires fewer animals, reduces variability inherent in comparing different animals, and is more economical both in terms of time and money compared with more traditional approaches for analysis of gene therapy methods because fewer tissue samples need to be processed for analysis. Of the tissue types evaluated, decline in transgene expression was most rapid in the skin (3-4d) and most stable in the liver (10-14d). Thus, the principal finding of this study is that transgene expression kinetics are highly tissue-dependent.

Biolistic gene transfer methods are standard for plants, but relatively fewer studies have explored this method of gene transfer in animals. One of the key parameters that we initially evaluated was the DLR, which represents the amount of DNA used for coupling to a set amount of gold microcarriers. We analyzed various DLRs over a range recommended by the manufacturer of the Helios gene gun. Despite clear differences in DNA-gold coupling as evidenced by our gel electrophoresis results, there was surprisingly little difference in transfer efficiency in vivo using DLRs of 4, 10, or 25 (Figure 2). A likely explanation for these results is that the gold microcarriers became saturated with bound plasmid DNA when DLRs of 4 or higher were used, though higher order complex formation resulting in clumping of the microcarriers was observed when DLR was raised to 25. Such clumping can be problematic in that it makes it difficult to apply a uniform coating of the discharge cartridges, potentially leading to inconsistent results. Hence, we chose the next highest DLR of 10 for our experiments to avoid this potential problem. These findings are consistent with previous reports [31] and with preliminary studies in our laboratory where we found that DLRs of 2 or lower appeared to produce less reporter gene expression compared to DLRs of 4 or higher (not shown). Conversely, using DLRs greater than 10 is probably not recommended because most of the DNA used will be uncoupled or bound up in higher order complexes resulting in clumping. Therefore, the optimal DLR appears to be in the range of 4-10 when using conditions employed in the present study.

Helium pressures used in our study varied between 200-400 psi. Although not systematically evaluated here, we found that pressures of 300-400 psi were well-tolerated in skin. Other studies have used even higher pressures (500 psi) for biolistic-mediated gene "vaccinations" in skin and also observed relatively little tissue damage [33,40,41]. Maximum recommended helium pressure for the Bio Rad Helios gene gun is 600 psi. It is anticipated that "tough" tissues such as skin and muscle can tolerate pressures approaching this maximum, but that "soft" internal tissues may not. Consequently, we used lower helium pressure (200 psi) for gene transfer to liver, which is similar to what was used in a previous study (250 psi) to deliver the DNA element regulating cytochrome P450 2B1 [32,42]. We were reluctant to try higher pressures in liver for fear of causing serious tissue damage, and there did not appear to be a need to do so anyway because expression in liver was fairly robust under the conditions utilized here. In fact, expression persisted in liver for several days beyond that observed in the skin. The reason for this observation is not clear, but may be due in part to relatively high turnover of epidermis and/or differential nuclease activities in the two tissues. Indeed, histological assessments revealed that most of the microcarriers were localized to the epidermis following biolistic transfer to skin, with a maximum penetration of not more than a few hundred microns representing approximately 20-30 cell layers from the surface even when employing the highest helium pressure (400 psi) used in this study. Taken together, these results suggest that biolistic gene transfer conditions need to be optimized for each different type of tissue targeted.

## Study Limitations and Future Directions

This investigation was limited to two target tissues in vivo: skin and liver. To compare expression from biolistic-delivered reporter genes, we shot the same CMV-LUC gold "bullets" into these tissues and compared the resulting responses over time in vivo using BLI. An advantage of using the CMV-LUC reporter is that it produces strong LUC activity that is readily measured using BLI. This appears to work particularly well for short-term transient expression, and is consistent with earlier work showing that this promoter is strongly expressed in mouse liver following transfection in vivo [43]. On the other hand, the CMV promoter/enhancer has limited and questionable utility for longer-term sustained transgene expression applications. Even though we did not use a viral vector, the presence of the strong viral enhancer/promoter is still a potential concern. It is well known, for example, that CMV and other strong viral promoter are typically silenced by host methylation mechanisms once they integrate into genomic DNA [44,45]. The site of integration can also be problematic in some cases such as those where nearby proto-oncogenes get activated as a result [14,15]. Future studies will be developed to investigate tissue-specific promoters tailored to the relevant target tissues. The present study focused on short-term transient expression, but future studies could evaluate more sustained expression over time for various gene therapy strategies. The use of homologous human sequences and tissue-specific non-viral enhancer/promoter elements could be directly applied using biolistic approaches. BLI should prove useful for continued evaluation of these approaches in near real-time in vivo.

## Conclusions

We have shown that biolistic gene transfer can be efficiently optimized in different tissues using non-invasive BLI to monitor expression in the same animals repeatedly over time in vivo. Of the representative tissue types evaluated, expression peaked within 2-3 days for both, but declined most rapidly in the skin (3-4 days) compared to liver (10-14 days). Thus, tissue-specific expression kinetics should be an important consideration in the design of effective gene therapies using physical gene transfer techniques, which may serve as potentially useful gene delivery strategies compared with existing viral-based approaches. Biolistic gene transfer methods appear to offer an attractive, safe, and effective alternative to viral vectors for gene therapeutic strategies that can be directly applied in the clinic to treat a wide variety of human ailments. So far, biolistic gene transfer applications in the clinical setting have been primarily focused on transfection of cells in culture which are then transplanted to the patient [46-49]. A more recent study [50] directly targeted external tissues such as epidermis for vaccination applications, and it is anticipated that there will be more of these types of applications developing for biolistic gene transfer in the future. The BLI-based assessment strategy described here should facilitate optimization of biolistic conditions for different tissue types in the pre-clinical setting, thereby providing an efficient means of pre-evaluation of in vivo efficacy in animal models prior to human trials.

## Authors' contributions

JX performed all of the experiments and produced the figures and primary drafts of this manuscript. AM assisted with the experiments and analysis of results. HD provided valuable insight and direction for the biolistic experiments and techniques used in this study. SNE directed the research, provided the major funding for the project, analyzed the results, and was responsible for the final editing and overall content of the manuscript. All authors have read and approved the final manuscript.
